# Association between somatic amplification, anxiety, depression, stress and migraine

**DOI:** 10.1186/1129-2377-14-53

**Published:** 2013-06-25

**Authors:** Burcu Goksan Yavuz, Elif Ilgaz Aydinlar, Pinar Yalinay Dikmen, Cem Incesu

**Affiliations:** 1 Department of Psychiatry, Acibadem University School of Medicine, Istanbul, Turkey; 2 Department of Neurology, Acibadem University School of Medicine, Istanbul, Turkey

**Keywords:** Migraine, Somatosensory amplification, Migraine disability, Depression, Anxiety, Stress

## Abstract

**Background:**

The aim of this study is to investigate the associations between migraine related disability and somatosensory amplification, depression, anxiety, and stress.

**Method:**

Fifty-five migraine patients who applied to the outpatient unit of the Neurology Department of Acibadem University School of Medicine, Maslak Hospital in Istanbul, Turkey, and twenty-eight subjects without migraine were recruited for the study. The participants were asked to complete a sociodemographic form, Migraine Disability Assessment Scale (MIDAS), Depression Anxiety Stress Scale, Somatosensory Amplification Scale (SSAS).

**Results:**

Somatosensory amplification scores were significantly higher in the migraineurs than in the control group (29.85+/−6.63 vs 26.07+/−7.1; p=0.027). Somatosensory amplification scores and depression scores were significantly higher in migraineurs with moderate and severe disability than in patients with minimal and mild disability (31.7+/−6.4 vs 27.71+/−5.49; p=0.01, 11.27+/−8.7 vs 7.38+/−8.11; p=0.04, respectively). A significant positive correlation was found between the frequency of migraine attacks for at least three consecutive months (MIDAS A scores) and the SSAS scores (r=0.363, p=0.007) in migraineurs. The MIDAS total scores were also significantly correlated with the DASS depression subcale scores (r=0.267, p=0.04), and the DASS stress subscale scores (r=0.268, p=0.05).

**Conclusion:**

Psychological factors, and vulnerability to bodily sensations may incease the burden of migraine. We point out that the timely assessing of somatic amplification and the evaluation of mental status would help improve the quality of life of in migraineurs.

## Background

Migraine, which is one of the leading neurological reasons for seeking medical care [1], is considered a chronic, disabling disease that significantly reduces quality of life [2,3]. The global prevalence of the adult members of the population with an active headache disorder are 46% for headache in general, 11% for migraine, 42% for tension-type headache and 3% for chronic daily headache [4]. In a nationwide epidemiological study in Turkey, the lifetime prevalence of migraine was found to be 10.9% in men and 21.8% in women [5].

Migraine has been reported to be associated with various well-known comorbidities [6]- conditions which occur in people with migraine with greater chance of frequency than in others [7]. Several studies suggest that mood and anxiety disorders are two to ten times more prevalent among people with migraine than in the general population, and greater than 25% of migraineurs meet the criteria for mood and anxiety disorders [8-10]. Population based studies demonstrate that depression is comorbid with migraine [11,12]. Major depression has been diagnosed in 40.7% of those with migraine and 16% of those in the control group [11]. In a study conducted by Lipton et al., 47% of migraineurs had depression while 17% of respondents in the nonmigraine group met the depression criteria, according to the Primary Care Evaluation of Mental Disorders screening questionnaire [12]. Other epidemiological studies have found that the lifetime prevalence of depression in migraine ranged from 17% to 42% [13-16]. A population-based study conducted by Chen et al. showed that 16% of the chronic migraine sufferers had anxiety disorders [17]. Breslau’s population based prospective study shows that 24.4% of the migraineurs suffered from anxiety disorders, and 13.5% had depression [18]. Breslau et al. found a greater than 4-fold risk of attempted suicide in migraineurs compared with controls who have never experienced headaches above mild intensity [18]. A longitudinal population based study shows that 30% of chronic migraine sufferers, and 19% of the patients with episodic migraine have anxiety [16]. Chronic stress is a widely recognized risk factor for both depression and chronic migraine [19]. It has been shown that psychological stress plays an important role not only before the onset of migraine [20,21], but also in the maintenance of the disorder, as well as the transformation from episodic to chronic migraine [22]. It is estimated that episodic migraine (EM) sufferers develop chronic migraine (CM) at the rate of 2.5% per year [23]. Previous studies have demonstrated that, when compared to EM, CM is associated with greater disability and lower quality of life [24-27]. Several studies support the view that psychiatric comorbidities can promote the transformation of episodic headaches into chronic daily headache [28,29]. This transformation may increase headache related disability, as well as the difficulty of treating it [13].

Although studies have demonstrated the association between migraine and psychiatric disorders, less is known about the relationship between somatic amplification and migraine related disability. Barsky et al. first introduced the notion of somatosensory amplification [30-32]. It was hypothesized that individuals who somatize perceive normal bodily sensations as unusually intense and disturbing. We hypothesize that somatic amplification together with depression, anxiety and stress may increase the burden of migraine and increase the risk of migraine related disability. Thus psychiatric comorbidity should not be underestimated, but should be treated promptly.

The present study was designed to determine the relationship between migraine related disability and somatic amplification and to consider its association with anxiety, depression and stress levels. To the best of our knowledge this is the fist study to investigate the relationship between the perception of bodily sensations and disability in migraineurs.

## Method

### Subjects

Fifty-five of ninety-four consecutive migraine patients aged between 15 and 50 years who applied to the outpatient unit of the Neurology Department of Acibadem University School of Medicine, Maslak Hospital in Istanbul, Turkey between September 2012 and January 2013 were recruited for the study. Migraine diagnosis was conducted by two neurologists according to the International Classification of Headache Disorders, second edition (ICHD-II) [33]. Exclusion criteria were the recent use of antidepressant and psychotropic drugs (“recent use” was determined as 8 weeks or less before the study since all known antidepressants studied in clinical trials cause a clinical response in about two-thirds of patients within 8 weeks of initiating treatment [34]), acute and chronic psychosis, mental retardation and illiteracy. Thirty-nine patients were excluded due to their recent use of psychotropic drugs or unwillingness to participate in the study. Twenty-eight healthy control subjects matched in age and sex were recruited at the same time. The control subjects did not meet the criteria for either migraine or non-migraine headache. Migraineurs were divided into two groups, CM and EM. To be classified as CM, a respondent had to report an average of 15 or more headache days per month within the past three months. EM was defined as reporting an average of 14 or fewer headache days per month within the past three months. In order to evaluate the sociodemographic and clinical characteristics of the participants, they were asked to answer a sociodemographic questionnaire. After they had been informed about the study and given written informed consent, the subjects were asked to fill out a Migraine Disability Assessment Scale (MIDAS), Depression Anxiety Stress Scale (DASS) and Somatosensory Amplification Scale (SSAS). All of the fifty five the subjects were found eligible and they all agreed to participate in the study. One patient with migraine did not fillout the migraine disability assessment scale.

The study protocol was conducted in accordance with the ethical principles stated in the “Declaration of Helsinki” and approved by the Ethical Committee of Acibadem University School of Medicine.

## Materials

### Sociodemographic data form

The sociodemographic form was developed by the investigators to evaluate sociodemographic characteristics such as age, sex, education status, marital status, occupational status and clinical characteristics.

### Migraine disabilty assessment scale

The disability associated with migraine was assessed using the validated Turkish version of MIDAS. This has five questions assessing the number of working days lost due to migraine over a three month period. MIDAS gathers information on migraine related disability in terms of work/study, household work, and leisure activities on the days when the headache was experienced [35]. The questions are asked regarding either days of missed activity or days during which productivity was reduced by at least 50%. The total days are summed and categorized into four grades of severity. The four point grading system for the MIDAS questionnaire is as follows: Grade I (scores ranging from 0 to 5), little or no disability; Grade II (scores ranging from 6 to 10), mild disability; Grade III (scores ranging from 11–20), moderate disability; Grade IV (scores of 21 or greater), severe disability. MIDAS A and MIDAS B assess headache frequency and pain intensity (0= no pain; 10= very severe pain) over a three month period. The Turkish version of the MIDAS questionnaire was developed by Ertas et al. [36].

### Somatosensory amplification scale

The Somatosensory Amplification Scale evaluates sensitivity to mild bodily sensations which are unpleasant and disturbing but non-pathological. This self-evaluating scale developed by Barsky et al. consists of 10 items that are estimated on a five-point scale ranging from 1 (‘not at all’) to 5 (‘extremely’) [31]. The statements describe a physical discomfort which does not indicate a disease. By summing up the scores a total amplification score is obtained. The reliability and validity of the Turkish form was established by Gulec et al. [37].

### Depression anxiety stress scale

The Depression Anxiety Stress Scale was used in the present study for data collection to assess the negative emotional symptoms among participants. It is a 42 item self-report inventory designed by Lovibond et al. [38]. It provides scores on three subscales: depression (14 items), anxiety (14 items) and stress (14 items). Each item is rated on a four-point Likert scale showing the frequency or severity of the participants’ experiences over the last week. This scale was validated to Turkish by Akin et al. [39].

### Statistical analyses

Statistical analyses were performed using the Statistical Package for the Social Sciences (SPSS) version 13.0 (SPSS Inc., Chicago, IL., U.S.A, 2005) The data of categorical variables were demonstrated as counts and percentages. The data of continuous variables were presented as the mean and standard deviation. The Mann Whitney U test was used to compare the nonparametric variables. The correlations between the scales were tested using Pearson’s Correlation Analysis. The level of statistical significance was set at p<0.05.

## Results

### Sociodemographic and clinical characteristics

A total of 55 migraineurs answered the questionnaires in this study. Of the 55 patients, 42 (76.4%) were female and 13 (23.6%) were male. The mean age of the participants was 31.98±7.22 (ranging between 15 and 47) years. 72.7% of the migraineurs were employed and 57.4% were married. Among the 55 migraineurs, 52 (94.5%) had migraine without aura, while the remaining 3 patients (5.5%) had migraine with aura. The mean duration of migraine was 8.78±6.8 years. 90.9% of the patients had accompanying symptoms such as photophobia, phonophobia, nausea and vomiting. Five of the 55 (9.3%) patients had vertigo. The mean pain intensity of the patients was 7.35±1.31 (numerical analogue scale, ranging between 0 and 10). The mean number of attacks for at least three consecutive months was 17.96±13.04 days. The mean duration of attacks was 1.66±0.81 days. Three patients (5.5%) suffered from CM Table 1 shows the sociodemographic and clinical characteristics.

**Table 1:** **Sociodemographic and clinical characteristics of migraineurs** (**n**=**55**)

***Characteristics***	***Migraineurs (n=55)***			***Control group(n=28)***		
	***Mean±SD***	***n***	**%**	***Mean±SD***	***n***	**%**
***Sex***						
Female		42	76.4		19	67.9
Male		13	23.6		9	32.1
Age	31.99±7.22			31.86±5.77		
***Migrane and associated symptoms***						
With aura		3	5.5			
Without aura		52	94.5			
Episodic		52	94.5			
Chronic		3	5.5			
Duration of migraine (years)						
Duration of attacks (days)	1.66±0.81					
Attacks per month	6.02±4.31					
Pain intensity (NAS)	7.35±1.31					
Nausea		40	72.7			
Vomitting		7	12.7			
Photophobia		43	78.2			
Phonophobia		26	47.3			
Vertigo		5	9.3			
***Previous psychiatric history***						
None		45	81.8		28	100
Depression		8	14.5			
Anxiety		2	3.6			

*SD* Standart deviation, n= number of subject, *NAS* Numeric analogue scale.

### Association between migraine related disability, somatic amplification, depression, anxiety and stress levels

The MIDAS scores showed that 13 patients (24.1%) had minimal disability, 8 (14.8%) patients had mild disability, 16 (29.6%) had moderate disability and 17 (31.5%) had severe disability (Table 2). The total scores for the migraine disability assessment scale were significantly higher in female patients than in male patients (38.6±6.03 vs 7.01±1.94; p=0.03). Somatosensory amplification scores and depression scores were significantly higher in migraineurs with moderate and severe disability than in patients with minimal and mild disability (31.7±6.4 vs 27.71±5.49; p=0.01, 11.27±8.7 vs 7.38±8.11;p=0.04, respectively) (Table 3).

**Table 2:** MIDAS grades of the migraineurs

**MIDAS grade**	**%**
**Grade I(little/no disability)(n=13)**	24.1
**Grade II(mild disability)(n=8)**	14.8
**Grade III(moderate disability)(n=16)**	29.6
**Grade IV(severe disability)(n=17)**	31.5

N= number of subject, MIDAS: Migraine diability assessment scale.

**Table 3:** **Comparison of scores between minimal**- **mild disability group and moderate** - **severe disability group**

**Scales**	**Group I (n=21) (MIDAS I-II) (Minimal-mild disability)**	**Group II(n=33)(MIDAS III-IV)(moderate-severe disability)**	**P**
**SSAS**	27.71±5.49	31.7±6.4	**0.01**
**DASS depression**	7.38±8.11	11.27±8.7	**0.04**
**DASS anxiety**	7.9±5.71	11.67±7.95	0.09
**DASS stress**	14.29±8.6	17.94±7.84	0.1

*MIDAS* Migraine disability assessment scale, *SSAS* Somatosensory amplification scale, *DASS* Depression anxiety stress scale, n=number of subject.

A significant positive correlation was found between the frequency of migraine attacks for at least three consecutive months (MIDAS A scores) and the SSAS scores (r=0.363, p=0.007) in migraineurs. The level of stress and anxiety symptoms was significantly associated with the presence of somatic amplification. Our analysis showed significant correlations between the somatic amplification scores and the scores for anxiety (r=0.413, p=0.002) and stress (r=0.291, p=0.03) (Table 4). The MIDAS total scores were also significantly correlated with the DASS depression subcale scores (r=0.267, p=0.04) and the DASS stress subscale scores (r=0,268, p=0.05) (Table 4).

**Table 4:** Correlation of DASS scores with MIDAS total scores and SSAS

	**DASS depression r p**	**DASS anxiety r p**	**DASS stress r p**
**MIDAS total**	r=0.276 **p**=**0**.**04**	r=0.165 p=0.23	r=0.268 **p**=**0**.**05**
**SSAS**	r=0.196 p=0.15	r=0.413 **p**=**0**.**002**	r=0.291 **p**=**0**.**03**

*DASS* Depression anxiety stress scale, *MIDAS* Migraine disability assessment scale, *SSAS* Somatosensory amplification scale.

According to the DASS scores 38.2% of the patients had depressive symptoms, 61.8% of them had anxiety symptoms and 52.7% of the patients had mild, moderate or severe stress levels. Table 5 shows the grading of DASS scores in the migraineurs.

**Table 5:** Prevalence of depression, anxiety and stres levels according to DASS

**DASS subscales**	**Normal N(%)**	**Mild N(%)**	**Moderate N(%)**	**Severe N(%)**	**Extremely severe N(%)**
**Depression**	34(61.8)	7(12.7)	7(12.7)	4(7.3)	3(5.5)
**Anxiety**	21(38.2)	8(14.5)	13(23.6)	7(12.7)	6(10.9)
**Stress**	27(49.1)	7(12.7)	10(18.2)	9(16.4)	2(3.6)

n=number of subject, *DASS* Depression anxiety stress scale.

Depression, anxiety and stress levels were significantly higher in the migraine patients than in the healthy control subjects: the Depression subscale (10.02±8.75 vs 6.96±8.72; p=0.03), the Anxiety Subscale (10.24±7.28 vs 6.96±8.72; p=0.03) and the Stress Subscale (16.51±8.2 vs 12.61±8.79; p=0.02). Somatosensory amplification scores were significantly higher in the magraineurs than in the control group (29.85±6.63 vs 26.07±7.1; p=0.027). Table 6 shows a comparison of the migraineurs’ scores and those of the control subjects. We also found that the scores in SSAS (36.0±4.0 vs 29.5 ±6.6; p=0.07), DASS depression (16.67±18.18 vs 9.63±8.1; p=0.5), anxiety (13.33±7.77 vs 10.06±7.3; p=0.4) and stress (24.0±7.98 vs 16.08±8.07; p=0.08) scales in patients with CM were higher than in patients with EM.

**Table 6:** Comparison of scores between migraineurs and healthy control subjects

**Scales**	**Migraineurs(n=55)Mean±SD**	**Control group(n=28)Mean±SD**	**P**
**SSAS**	29.85±6.63	26.07±7.1	**0**.**027**
**DASS**-**depression**	10.02±8.75	6.96±8.72	**0**.**03**
**DASS**-**anxiety**	10.24±7.28	7.79±8.27	**0**.**03**
**DASS**-**stress**	16.51±8.2	12.61±8.79	**0**.**02**

*SSAS* Somatosensory amplification scale, *DASS* Depression anxiety stress scale.

## Discussion

The present study investigated the association between migraine disability and somatic amplification in a sample of 55 migraineurs. It also evaluated the relationship between migraine disability and psychological distress.

Our findings show that migraineurs had a significantly higher than average tendency to be aware of bodily sensations. Somatic amplification scores were significantly higher in migraine patients than in control subjects. Our results also point out the role of somatic amplification in migraine related disability. According to our data migraineurs with moderate and severe migraine related disability have higher somatosensory amplification scores than the patients with minimal and mild disability. Moreover there was a significant positive correlation between the SSAS scores and the frequency of headache for the last three months. Patients who have an increased attention to unpleasant bodily sensations and the tendency to appraise vague somatic sensations as abnormal or pathological have more migraine attacks which may lead to a lower quality of life.

Psychological distress is well kown to be associated with increased physical symptoms [40]. Similarly our findings showed significant correlations between somatic amplification and level of anxiety and stress. Maizels et al. revealed that the increased prevalence of somatic symptoms in headeache patients with psychiatric comorbidity is not suprising [41], and somatoform disorders were diagnosed in the range 6%-22% of patients with chronic daily headache [42,43]. Researchers have shown that depression and anxiety disorders are frequently reported in adults with migraine [8-17]. In this study 38.2% of the migraineurs suffered from depressive symptoms. Similarly previous studies showed that the incidence of depressive symptoms ranges from 17% to 42% [13-16]. Our data shows that 62% of the patients with migraine had anxiety symptoms. This finding was higher than the rates reported in studies from Italy (38.1%) [44], China (38.1%) [45] and the USA (51%) [46].

Consistent with Jelinkski’s study, [47] we found that migraine related disability was significantly correlated with the level of depressive symptoms. In our study the severity of stress was also significantly correlated with migraine related disability. Our finding supports the necessity of treatment for psychiatric disorders in migraine patients since migraine and psychological distress significantly reduce the quality of life [13] and lead to a worse prognosis, chronic disease and a reduced response to treatment [48]. It has been indicated that migraine comorbid with depession or anxiety causes higher medical costs than does migraine alone [49].

Psychological factors and vulnerability to bodily sensations may incease the burden of migraine. Some episodic migraine patients develop chronic migraine through a transformative process [23,50,51]. Chronic migraine accounts for approximately 50% of all chronic daily headaches in the general population [52]. Poplation-based studies have demonstrated that compared to EM, CM is associated with greater migraine-disability, reduced quality of life, increased medical and psychiatric comorbidities, increased risk of medication overuse [16,23,50]. Therefore as Yong et al. say early diagnosis and treatment for psychiatric comorbidiy are very important [45]. Yong et al. suggest that if comorbid depression is not recognized and treated effectively, succesful headache management is unlikely [45]. Thus clinicians should be alert to psychological stress and somatic amplification when evaluating migraine patients.

Among our findings, we point out that the timely assessing of somatic amplification and the evaluation of psychological status would prevent migraine from becoming chronic and turning into a headache from medication overuse. Thus this strategy would help improve the quality of life of in migraineurs.

## Conclusion

Previous studies have examined the psychiatric comorbidity and somatic symptoms in migraine patients. To the best of our knowledge this is the first study to investigate the association between somatic amplification and migraine disability. However our study has some limitations. First, our sample size was small and this may prevent our results from being generalized. Moreover we evaluated psychological distress only by a self-reported questionnaire. Future studies with a large sample size and a detailed psychiatric evaluation are needed to investigate further the influence of somatic amplification and psychological distress in migraine related disability.

## Competing interests

The authors declare that they have no competing interests.

## Authors’ contributions

BGY, EIA, PY, CI participated in the study and drafted the manuscript. All authors read and approved the final manuscript.
